# Secondary Phase Interaction at Interfaces of High-Strength Brazed Joints made using Liquid Phase Sintered Alumina Ceramics and Ag-Cu-Ti Braze Alloys

**DOI:** 10.1038/s41598-018-20674-w

**Published:** 2018-02-20

**Authors:** T. A. Kassam, N. Hari Babu, N. Ludford, S. Yan, A. Howkins

**Affiliations:** 1 Brunel Centre for Advanced Solidification Technology (BCAST) Brunel University London, Uxbridge, UB8 3PH UK; 2 Specialist Materials and Joining (SMJ), TWI Ltd, Granta Park, Cambridge, CB21 6AL UK; 3 Loughborough Materials Characterisation Centre (LMCC), Loughborough University, Loughborough, LE11 3TU UK; 4 Experimental Techniques Centre (ETC), Brunel University London, Uxbridge, UB8 3PH UK

## Abstract

Alumina-to-alumina brazed joints were formed using 96.0 and 99.7 wt.% Al_2_O_3_ ceramics using 150 µm thick Ticusil^®^ (68.8Ag-26.7Cu-4.7 wt.% Ti) braze preforms. Brazing was conducted in a vacuum of 1 × 10^−5^ mbar at 850 °C for 10 minutes. Joint strengths were evaluated using four-point bend testing and were compared to the monolithic flexural strengths of standard alumina test bars according to ASTM C1161-13. Brazed joints made using 96.0 wt.% Al_2_O_3_ consistently outperformed brazed joints made using 99.7 wt.% Al_2_O_3_, despite similarities in both the flexural strengths of the standard alumina test bars and the microstructures of brazed joints. Secondary phase interaction led to the formation of Ti_5_Si_3_ reaction products at locations where the triple pocket grain boundaries of the 96.0 wt.% Al_2_O_3_ surface intersected the Ti-rich reaction layers. It is proposed that due to this interaction, brazed joints made using 96.0 wt.% Al_2_O_3_, which were relatively cost-effective to produce, achieved higher strengths than brazed joints made using 99.7 wt.% Al_2_O_3_.

## Introduction

Polycrystalline alumina has been the most commonly used ceramic material in joining studies^1^. A range of ceramic-to-metal joining techniques^2–4^ have been used to exploit the refractoriness, electrical insulation, wear- and corrosion-resistance properties of alumina. One of the industrially preferred methods for joining ceramics is brazing, whereby a braze alloy is melted between the surfaces of a ceramic material and another suitable material at elevated temperatures (>450 °C). The two most critical challenges in this process are; the poor wetting of chemically inert ceramic surfaces by metallic braze alloys, and thermally induced residual stresses generated due to a coefficient of thermal expansion mismatch at the joint interfaces.

Active metal brazing is a single-step liquid-state joining process (usually conducted in a vacuum atmosphere), whereby a braze alloy that contains a highly reactive element such as Ti, Hf or Zr, can wet an otherwise chemically inert ceramic surface^5^. The most commonly used active braze alloys are based on the Ag-Cu-Ti ternary alloy system, such as the commercially available braze alloy Ticusil®, with composition 68.8Ag-26.7Cu-4.5Ti wt.%.

In the established alumina/Ag-Cu-Ti system, often used as a model in the development of other ceramic/braze systems^6–10^, the joint interface typically consists of a nm-thick TiO layer on the alumina side of the joint interface and a µm-thick Ti_3_(Cu+Al)_3_O layer on the braze side of the joint interface^11–17^. The M_6_O-type reaction layer adds a higher degree of metallic character to the ceramic surface^18^, enabling the braze alloy to wet and spread effectively. The exact composition of the reaction layer can depend on the chemical activity of Ti, which can be influenced by the relative Ag, Cu, and Ti concentrations in the braze alloy, as well as the brazing temperature. Hence, several studies have reported the formation of either a single TiO layer^11,19–21^ at relatively lower Ti activity levels, or a single M_6_O-type Ti_4_(Cu+Al)_2_O layer^6,10,11^ at relatively higher Ti activity levels, at the joint interface. Additional chemical interactions associated with the dissolution of a parent metal, as in alumina-to-metal brazed joints made using Ag-Cu-Ti braze alloys, can also influence the chemical activity of Ti and its diffusivity towards the joint interfaces^8,22,23^.

The strengths of alumina-to-alumina brazed joints made using Ag-Cu-Ti braze alloys have previously been correlated to the thickness of the reaction layer, which can provide a good indication of chemical bonding at the joint interface^24,25^. Since the reaction layer is usually composed of a brittle phase, however, if excessively thick, degradation in joint strength can also occur. The thickness of the Ag-Cu braze interlayer (assuming complete diffusion of Ti to the joint interfaces) has also been found to influence the strength of alumina-to-alumina brazed joints^26–28^. The braze interlayer is the only ductile constituent in the alumina/Ag-Cu-Ti system which can plastically deform to accommodate thermally induced residual stresses. Hence, the residual stress state of a brazed joint can strongly affect the maximum stress that a brazed joint may withstand during mechanical testing and in-service.

Recently, excess Ti in the braze interlayer in the form of isolated Cu_4_Ti and Cu_4_Ti_3_ compounds was found to significantly improve the strength of alumina-to-alumina brazed joints^28^. This resulted from a controlled increase in the Ticusil braze preform thickness, which led to corresponding increases in both the reaction layer and braze interlayer thicknesses. In the same study, Kassam *et al*. (2015)^28^ found that joints made using 96.0 wt.% Al_2_O_3_ consistently outperformed those joints made using 99.7 wt.% Al_2_O_3_, despite similarities in both the flexural strengths of standard alumina test bars and the microstructures of the brazed joints.

**Figure 1:**
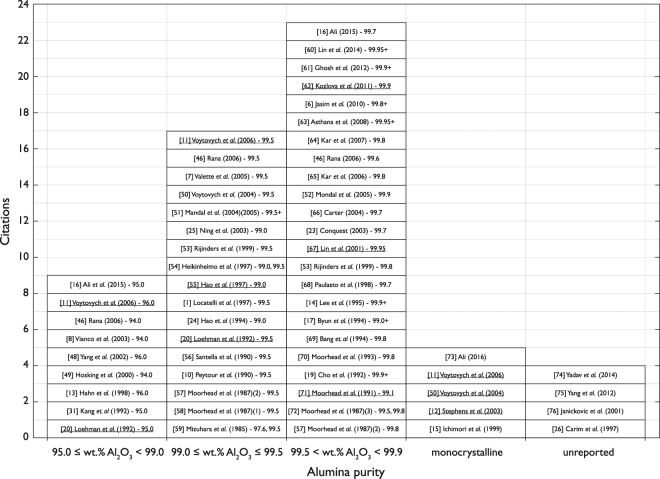
A summary of the grades of alumina selected in wetting experiments and in the formation of alumina-to-alumina brazed joints, using Ag-Cu-Ti braze alloys^46–76^. Underline denotes wetting experiments as opposed to the formation of alumina-to-alumina brazed joints. +denotes alumina ceramics were processed in-house, according to the following: Cho *et al*. (1991)^19^ – 99.9 % pure alumina powder with 1500 ppm MgO, sintered at 1600 °C for 2.0 hours. Byun *et al*. (1994)^17^ – polycrystalline alumina containing 1 % silicate. Lee *et al*. (1995)^14^ – 99.9 % pure alumina powder with 1000 to 1500 ppm MgO addition. Mandal *et al*. (2004) (2005)^51^ – 99.5 % pure alumina powder sintered at 1600 °C for 0.5 hours. Asthana *et al*. (2008)^63^ – 99.95 % pure alumina powder with 0.05 % MgO, sintered between 1200 to 1600 °C for 0.5 to 4.0 hours. Jasim *et al*. (2010)^6^ – 99.8 % pure alumina powder sintered at 1600 °C. Ghosh *et al*. (2012)^61^ – 99.99 % pure alumina powder sintered at 1600 °C for 2.0 hours. Lin *et al*. (2014)^60^ – 99.95 % pure alumina powder with 0.05 % MgO, sintered between 1200 to 1600 °C for 0.5 to 4.0 hours.

In the literature, most studies have opted to use solid-state sintered alumina ceramics, composed of at least 99.0 wt.% Al_2_O_3_ when performing brazing experiments using Ag-Cu-Ti braze alloys (Fig. 1). Less than ~20% of studies have selected liquid phase sintered alumina ceramics, whilst in other studies the alumina purity has not been reported. The superior mechanical properties of solid-state sintered alumina ceramics in comparison to liquid phase sintered alumina ceramics^29^ may have influenced these selections. The effect of secondary phase elements, present in liquid phase sintered alumina ceramics, on the interfacial chemistry and resulting joint strength, could not be found in the literature. This formed the basis of the investigation in this study.

## Results and Discussion

**Figure 2:**
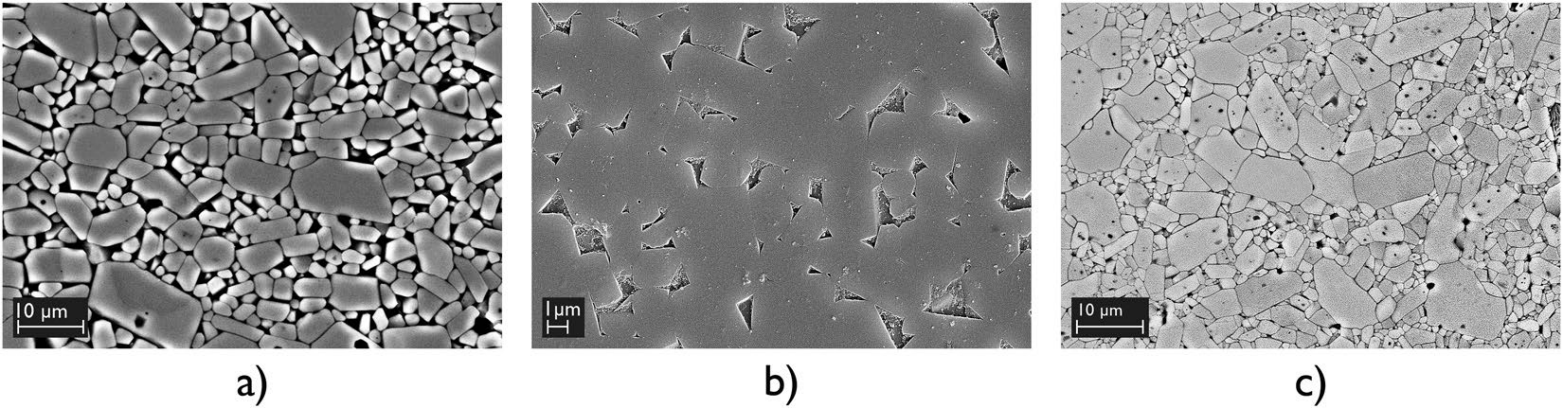
(**a**) Backscattered electron image of 96.0 wt.% Al_2_O_3_ polished and thermally etched at 1550 °C for 15 minutes, (**b**) Secondary electron image of 96.0 wt.% Al_2_O_3_ polished and chemically etched in 10 vol.% hydrofluoric acid-aqueous solution for 20 seconds, which revealed the secondary phase distribution, and (**c**) Backscattered electron image of 99.7 wt.% Al_2_O_3_ polished and thermally etched at 1550 °C for 15 minutes.

In this study, the liquid phase sintered 96.0 wt.% Al_2_O_3_ selected was found to contain 3.2 wt.% SiO_2_ as the main secondary phase, as well as 0.55 wt.% MgO and 0.06 wt.% CaO. The solid-state sintered 99.7 wt.% Al_2_O_3_ selected was found to contain 0.30 wt.% SiO_2_, 0.03 wt.% MgO and 0.02 wt.% CaO (Fig. 2). This phase analysis was performed using electron probe microanalysis, with average values based on 10 measurements.

**Figure 3:**
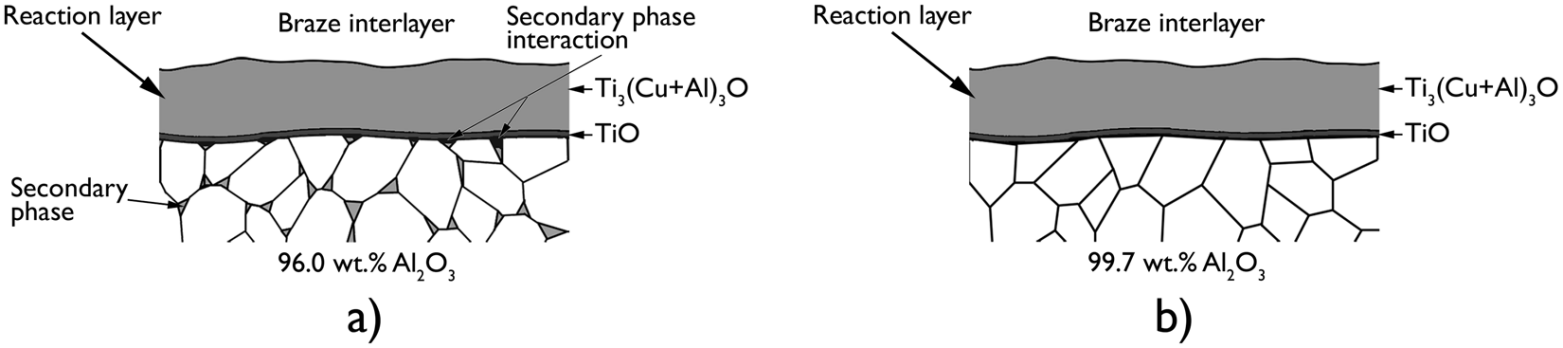
Schematics of the interfaces of brazed joints made using (**a**) 96.0 wt.% Al_2_O_3_, and (**b**) 99.7 wt.% Al_2_O_3_. Secondary phase interaction occurred at the interfaces of brazed joints made using 96.0 wt.% Al_2_O_3_ at locations where the triple pocket grain boundary regions of the alumina surface intersected with the Ti-rich reaction layers.

**Figure 4:**
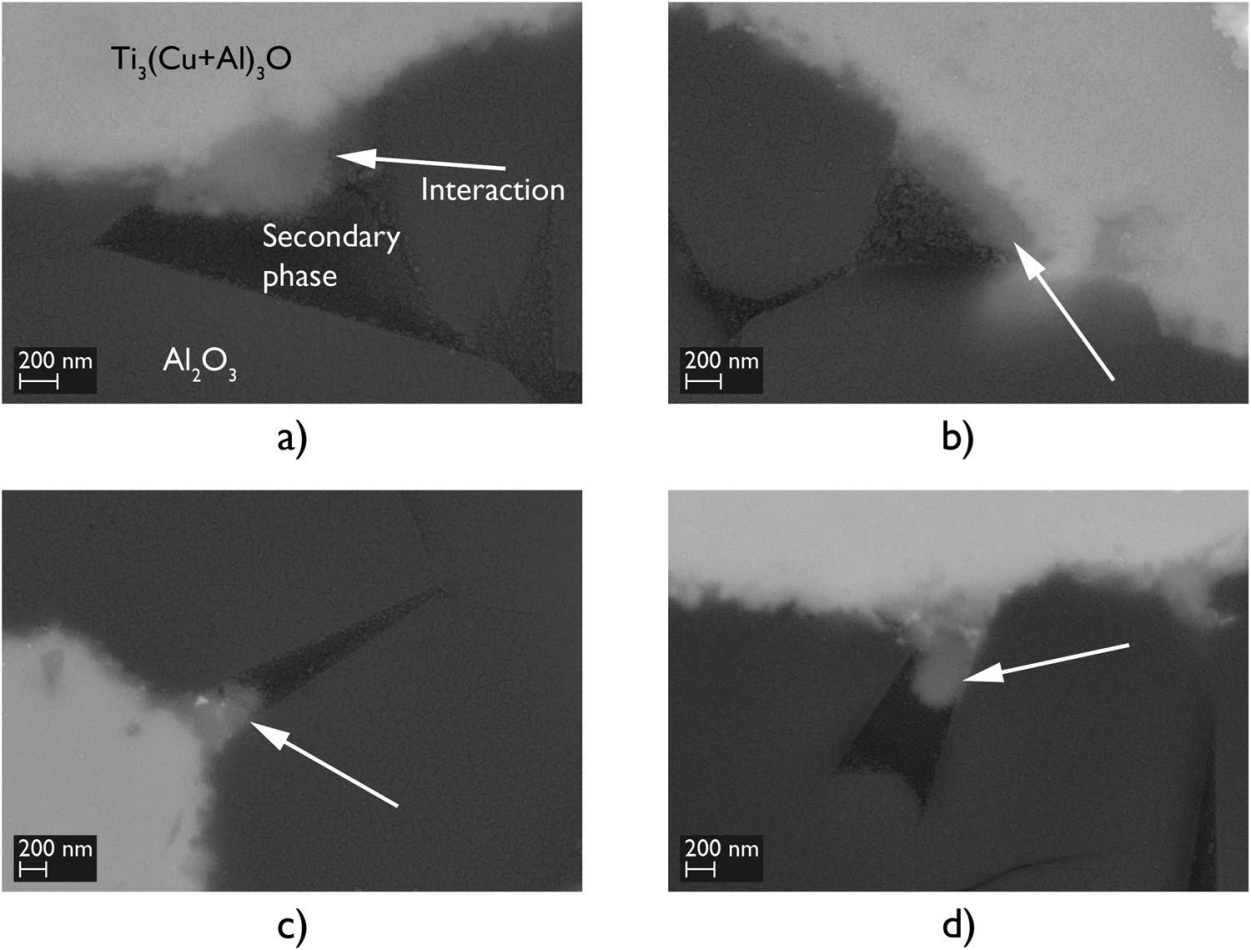
Backscattered electron images showing the secondary phase interaction (white arrows) observed in brazed joints made using 96.0 wt.% Al_2_O_3_ and Ticusil, at locations where the triple pocket grain boundary regions of the alumina surface intersected with the Ti-rich reaction layers.

The presence of Si at the interfaces of the alumina-to-alumina brazed joints made using 96.0 wt.% Al_2_O_3_ was observed, using SEM-EDX techniques, at locations where the triple pocket grain boundary regions of the alumina surface intersected with the Ti-rich reaction layers; beneath a γ - TiO layer on the alumina side of the joint interface (Fig. 3). These locations, characterised using backscattered electron imaging, showed the formation of a relatively darker phase in comparison to the Ti_3_(Cu+Al)_3_O layer which formed on the braze side of the joint interface. These results appeared to show that Ti and/or Cu from the braze alloy had penetrated approximately 300 to 500 nm deep into the triple pocket grain boundary regions of the faying surfaces of 96.0 wt.% Al_2_O_3_, where these elements had interacted with the relatively lower atomic number elements Si and/or Mg. This secondary phase interaction was observed at every location where the triple pocket grain boundary regions of the 96.0 wt.% Al_2_O_3_ surface intersected with the Ti-rich reaction layers (Fig. 4). No such observation was made at the interfaces of the alumina-to-alumina brazed joints made using 99.7 wt.% Al_2_O_3_.

**Figure 5:**
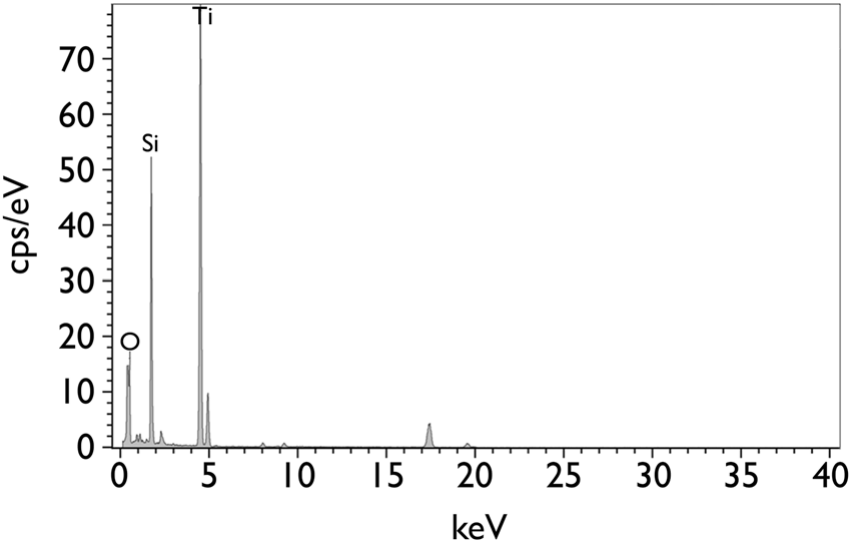
Scanning transmission electron microscopy-Energy dispersive X-ray analysis spectrum acquired from Ti_5_Si_3_, corresponding to Fig. 6.

**Figure 6:**
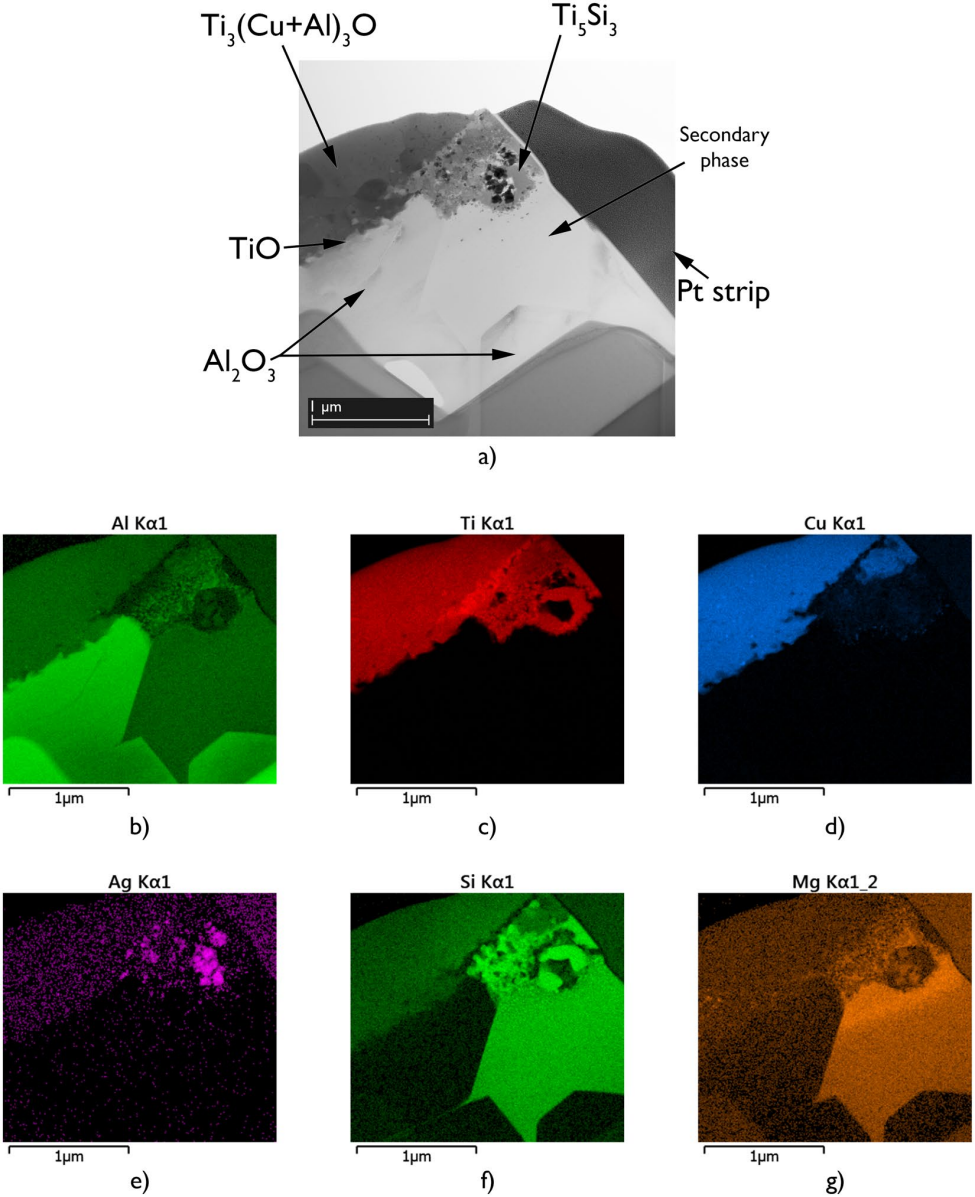
(**a**) Bright field scanning transmission electron microscopy image of a typical region at the interface of a brazed joint made using 96.0 wt.% Al_2_O_3_ and Ticusil, showing the formation of Ti_5_Si_3_ at a location where the triple pocket grain boundary region of the alumina surface intersected with the Ti-rich reaction layers and, corresponding energy dispersive X-ray maps showing distributions of (**b**) Al, (**c**) Ti, (**d**) Cu, (**e**) Ag, (**f**) Si, and (**g**) Mg.

Using an *in-situ* lift out method performed using focussed ion beam milling, TEM specimens were prepared from locations of the interfaces of joints made using 96.0 wt.% Al_2_O_3_, where secondary phase interaction had been observed. STEM-EDX analysis indicated that the secondary phase interaction had led to the formation of Ti_5_Si_3_, with the composition of 67.4Ti-26.9Si-5.7O wt.% (Fig. 5). These Ti_5_Si_3_ compounds were observed to have formed between a triple pocket secondary phase grain, with composition 25.6Si-10.0Al-7.0Mg-57.4O wt.%, and the Ti-rich reaction layers; γ - TiO and Ti_3_(Cu+Al)_3_O. The resulting Ti_5_Si_3_ compounds, which appeared to have formed following the partial consumption of both the secondary phase grains and the Ti-rich reaction layers, were dispersed in an area that was approximately 1.0 µm wide. At the centre of this interaction region, several Ag particles that were 30 to 60 nm in diameter were also observed (Fig. 6).

**Figure 7:**
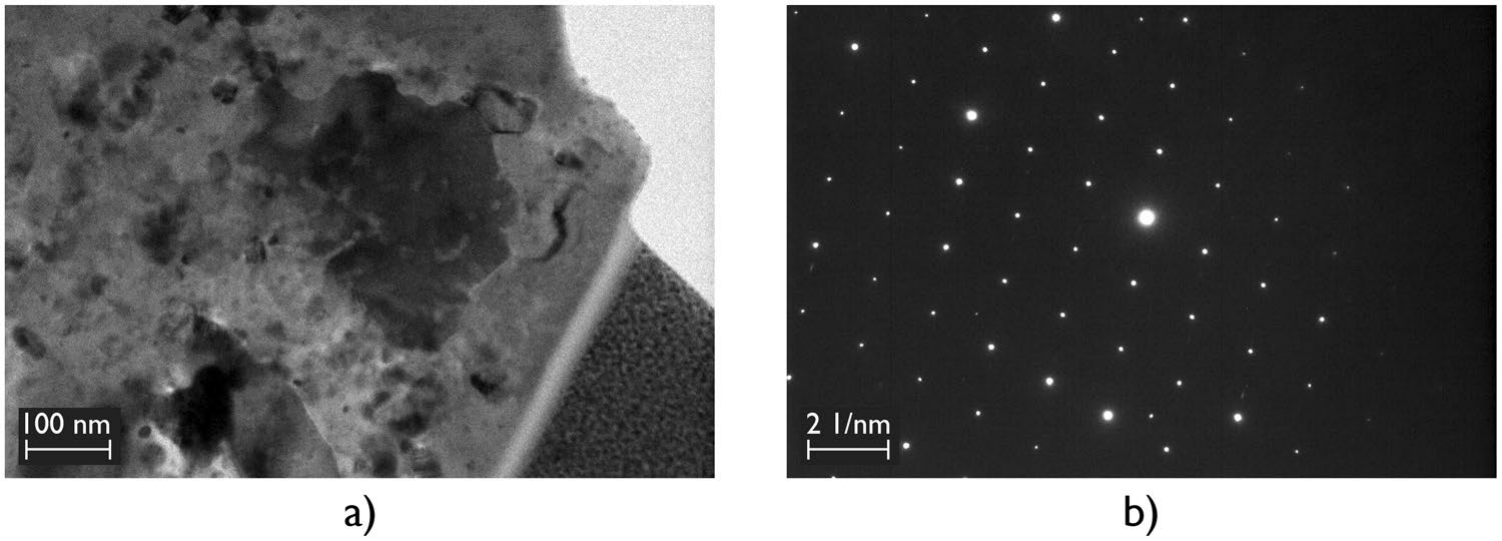
(**a**) High magnification bright field transmission electron microscopy image of a selected Ti_5_Si_3_ crystal and, (**b**) The corresponding selected area diffraction pattern acquired with zone axes of [100].

Electron diffraction was used to confirm the hexagonal Ti_5_Si_3_ phase of the P63/mcm (193) space group with lattice parameters a = b = 7.42 Å and c = 5.15 Å (Fig. 7). This was consistent with the selected area diffraction patterns of the Ti_5_Si_3_ phase, which have been reported elsewhere in the literature^30^.

**Figure 8:**
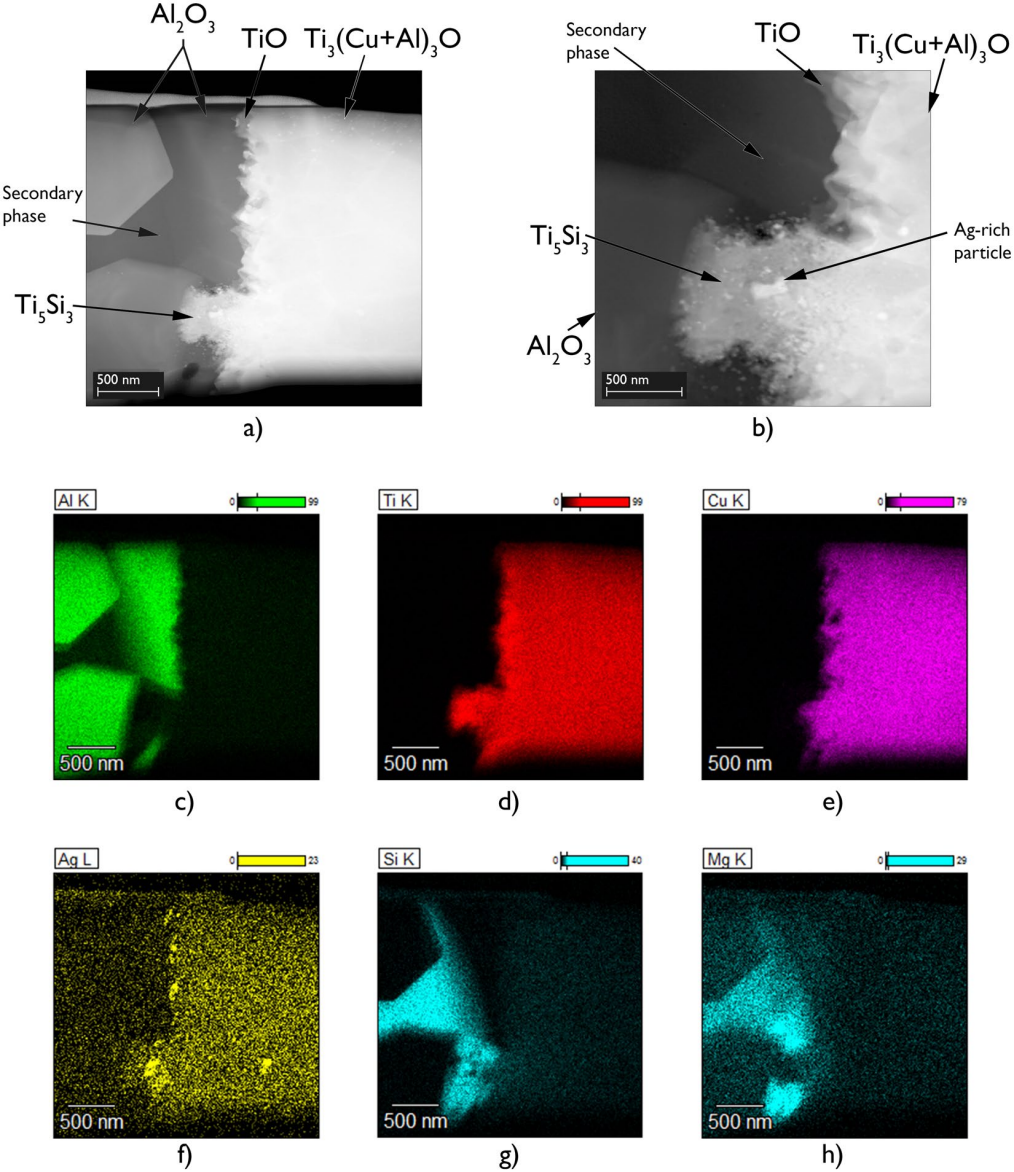
High angle angular dark field scanning transmission electron microscopy images of a typical region at the interface of a brazed joint made using 96.0 wt.% Al_2_O_3_ and Ticusil showing the formation of Ti_5_Si_3_ at a location where the triple pocket grain boundary region of the alumina surface intersected with the Ti-rich reaction layers, (**a**) Lower magnification, and (**b**) Higher magnification, and corresponding energy dispersive X-ray maps showing distributions of (**c**) Al, (**d**) Ti, (**e**) Cu, (**f**) Ag, (**g**) Si, and (**h**) Mg.

A second TEM specimen from a similar interfacial region of another brazed joint made using 96.0 wt.% Al_2_O_3_ showed a similar result i.e. secondary phase interaction. Again, the formation of Ti_5_Si_3_, with composition 74.1Ti-23.3Si-2.6O wt.% was observed at locations where the triple pocket grain boundary regions of the 96.0 wt.% Al_2_O_3_ surface intersected with the Ti-rich reaction layers (Fig. 8). At the centre of the 0.5 µm wide interaction region, small Ag particles were again observed.

The results confirmed the formation of Ti_5_Si_3_ at the interfaces of brazed joints made using 96.0 wt.% Al_2_O_3_ and Ticusil. This may have formed due to the presence of SiO_2_ at the triple pocket grain boundary regions of the 96.0 wt.% Al_2_O_3_ surface reacting with Ti, following the diffusion of Ti to the joint interfaces. A possible reaction mechanism is described in equations (1) and (2). It is postulated that following the reduction of SiO_2_ by Ti, the liberation of Si and its subsequent reaction with Ti led to the formation of Ti_5_Si_3_. These results were similar to the formation of Ti_5_Si_3_ reported in a study by Kang and Selvarian^31^, in which a 3 µm thick Ti coating was deposited onto the surface of a 95.0 wt.% Al_2_O_3_ ceramic before the coated alumina specimen was heated in a vacuum atmosphere to 980 °C for 10 minutes, simulating a typical brazing experiment.

1 $${\boldsymbol{Si}}{{\boldsymbol{O}}}_{{\bf{2}}}+{\bf{2}}{\boldsymbol{Ti}}\to {\boldsymbol{Si}}+{\bf{2}}{\boldsymbol{TiO}}$$

2 $${\bf{3}}{\boldsymbol{Si}}+{\bf{5}}{\boldsymbol{Ti}}\to {\boldsymbol{T}}{{\boldsymbol{i}}}_{{\bf{5}}}{\boldsymbol{S}}{{\boldsymbol{i}}}_{{\bf{3}}}$$

**Figure 9:**
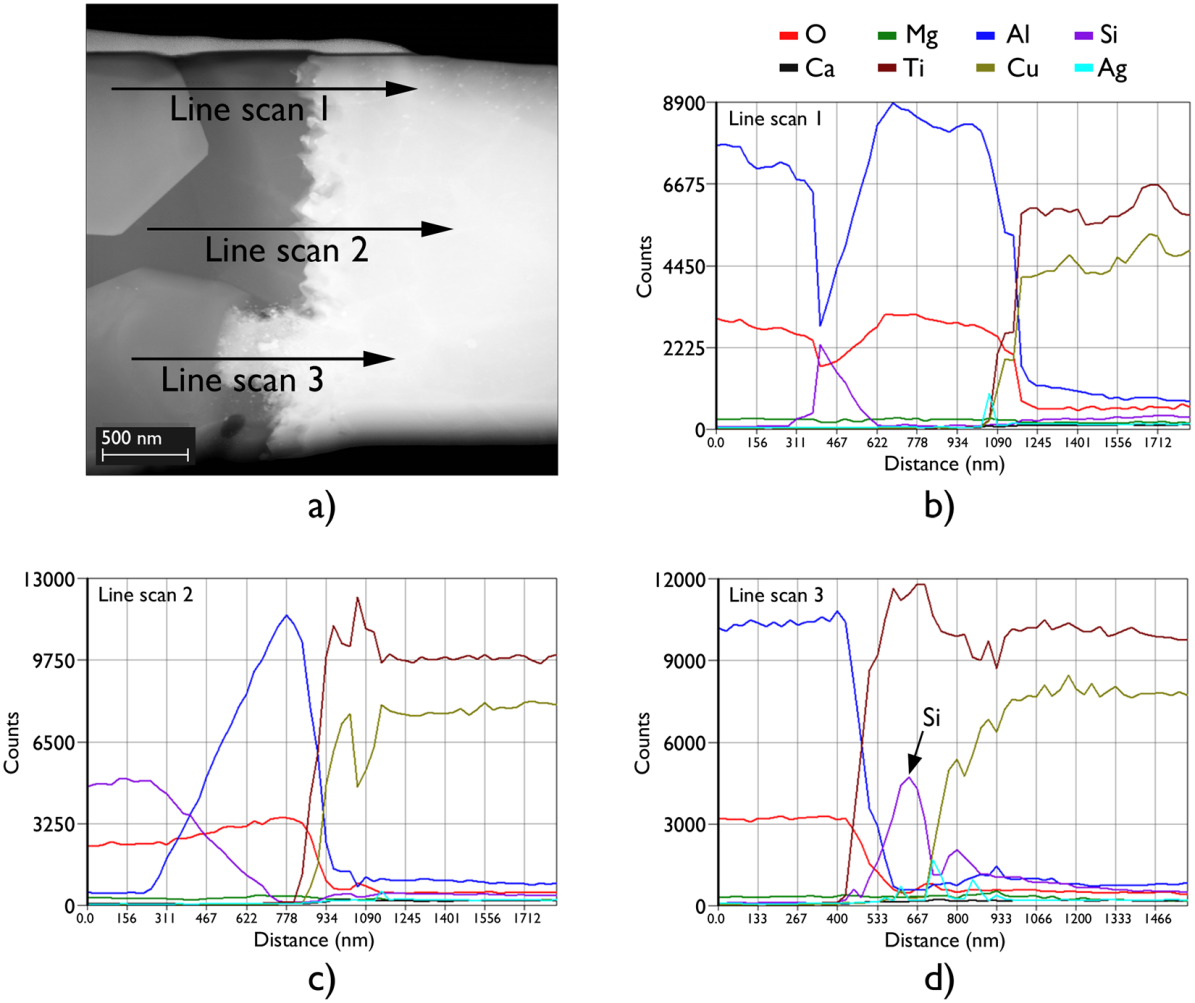
(**a**) High angle angular dark field scanning transmission electron microscopy image of a typical region at the interface of a brazed joint made using 96.0 wt.% Al_2_O_3_ and Ticusil, where secondary phase interaction led to the formation of Ti_5_Si_3_, (**b**) and (**c**) Line scans 1 and 2 passed through the secondary phase grain, however, since this grain was separated from the TiO and Ti_3_(Cu+Al)_3_O reaction layers by an alumina grain, Ti_5_Si_3_ did not form, (**d**) Line scan 3 passed through Ti_5_Si_3_, which formed where the secondary phase grain was in contact with the TiO and Ti_3_(Cu+Al)_3_O reaction layers.

The formation of Ti_5_Si_3_ has previously been reported to occur at the interfaces of joints made using both SiC^32,33^ and Si_3_N_4_^32,34–41^ ceramics and Ti-containing active braze alloys. In these systems, a Ti_5_Si_3_ layer can typically form as a result of either free Si on the ceramic surface, or from Si that is liberated following the reduction of the SiC or Si_3_N_4_ ceramic surface by Ti. In both of these systems, preventing the formation of a Ti_5_Si_3_ layer on the ceramic side of the joint interface (which can act as a diffusion barrier to Ti), whilst also preventing the diffusion of Si into the braze interlayer (which can lead to brittle reaction products), have been reported to help minimise any degradation in joint strength^41,42^. The formation of a Ti_5_Si_3_ layer in these systems, therefore, has typically been found to adversely affect joint strength. In this study, however, Ti_5_Si_3_ was not observed as a continuous layer at the joint interface but instead as 0.5 to 1.0 µm wide interaction regions. The formation of Ti_5_Si_3_ was observed only at locations where the triple pocket grain boundary regions of the 96.0 wt.% Al_2_O_3_ surface intersected with the Ti-rich reaction layers (Fig. 9). At these locations, the Ti_5_Si_3_ compounds did not appear to act as a diffusion barrier to Ti. Further, Si was not found to have diffused into the braze interlayer.

In addition to the γ - TiO and Ti_3_(Cu+Al)_3_O layers which were observed at the interfaces of brazed joints made using both 96.0 and 99.7 wt.% Al_2_O_3_ ceramics, the interfaces of brazed joints made using 96.0 wt.% Al_2_O_3_ also contained Ti_5_Si_3_, whereas the interfaces of brazed joints made using 99.7 wt.% Al_2_O_3_ did not. The absence of Ti_5_Si_3_ in brazed joints made using 99.7 wt.% Al_2_O_3_, therefore, was likely to have been due to the lack of any significant secondary phase in this solid-state sintered alumina.

The average flexural strengths of 96.0 and 99.7 wt.% Al_2_O_3_ standard test bars, following four-point bend testing according to configuration c of ASTM C1161-13, were 251.7 ± 4.8 MPa and 249.7 ± 3.8 MPa respectively. Brazed joints made using 96.0 wt.% Al_2_O_3_ and 150 µm thick Ticusil braze preforms achieved an average joint strength of 269.9 ± 9.2 MPa with failures that consistently occurred in the ceramic, away from the joint interface (Table 1). Despite both the presence of similar reaction layer thicknesses and brazed joint thicknesses, brazed joints made using 99.7 wt.% Al_2_O_3_ and 150 µm thick Ticusil braze preforms achieved an average joint strength of 212.1 ± 7.0 MPa, significantly lower than the 96.0 wt.% Al_2_O_3_ brazed joints. Mixed-mode failures were observed, occurring both in the 99.7 wt.% Al_2_O_3_ ceramic and at the joint interface.

**Table 1:** Average flexural strengths of 96.0 and 99.7 wt.% Al_2_O_3_ ceramics, and corresponding properties of brazed joints made using 150 µm thick Ticusil braze preforms.

Alumina purity, (wt.% Al_2_O_3_)	Average Flexural Strength (MPa)	Joint strength (MPa)	Braze preform thickness (µm)	Reaction layer thickness (µm)	Average brazed joint thickness (µm)	Specimens mechanically tested	Failure location
96.0	251.7 ± 4.8	269.9 ± 9.2	150	2.8 ± 0.1	65.3 ± 1.6	4	Ceramic
99.7	249.7 ± 3.8	212.1 ± 7.0	150	3.0 ± 0.1	67.8 ± 1.6	4	Mixed

The superior performance of brazed joints made using 96.0 wt.% Al_2_O_3_, in comparison to brazed joints made using 99.7 wt.% Al_2_O_3_, did not appear to be correlated to the reaction layer thickness or the braze interlayer thickness. Brazed joints made using 96.0 wt.% Al_2_O_3_ had an average reaction layer thickness of 2.8 ± 0.1 µm and an average brazed joint thickness of 65.3 ± 1.6 µm. Similarly, brazed joints made using 99.7 wt.% Al_2_O_3_ had an average reaction layer thickness of 3.0 ± 0.1 µm and an average brazed joint thickness of 67.8 ± 1.6 µm.

The bulk microstructures of both of sets of joints, made using 150 µm thick Ticusil braze preforms, were also similar. The braze interlayer in these joints consisted of Ag-rich and Cu-rich phases in a eutectic-like distribution, as well as several Cu-Ti phases in central regions. These Cu-Ti phases formed as a result of excess solid Ti in the braze interlayer, following the formation of stable γ - TiO and Ti_3_(Cu+Al)_3_O layers at the joint interfaces^28^.

The only significant difference between the brazed joints made using these two grades of alumina, which were both ground in the same way according to ASTM C1161-13, was the presence of Ti_5_Si_3_ at the interfaces of joints made using 96.0 wt.% Al_2_O_3_. This may have, therefore, provided the improvement in joint strength observed, which could be explained based on two possible mechanisms.

**Figure 10:**
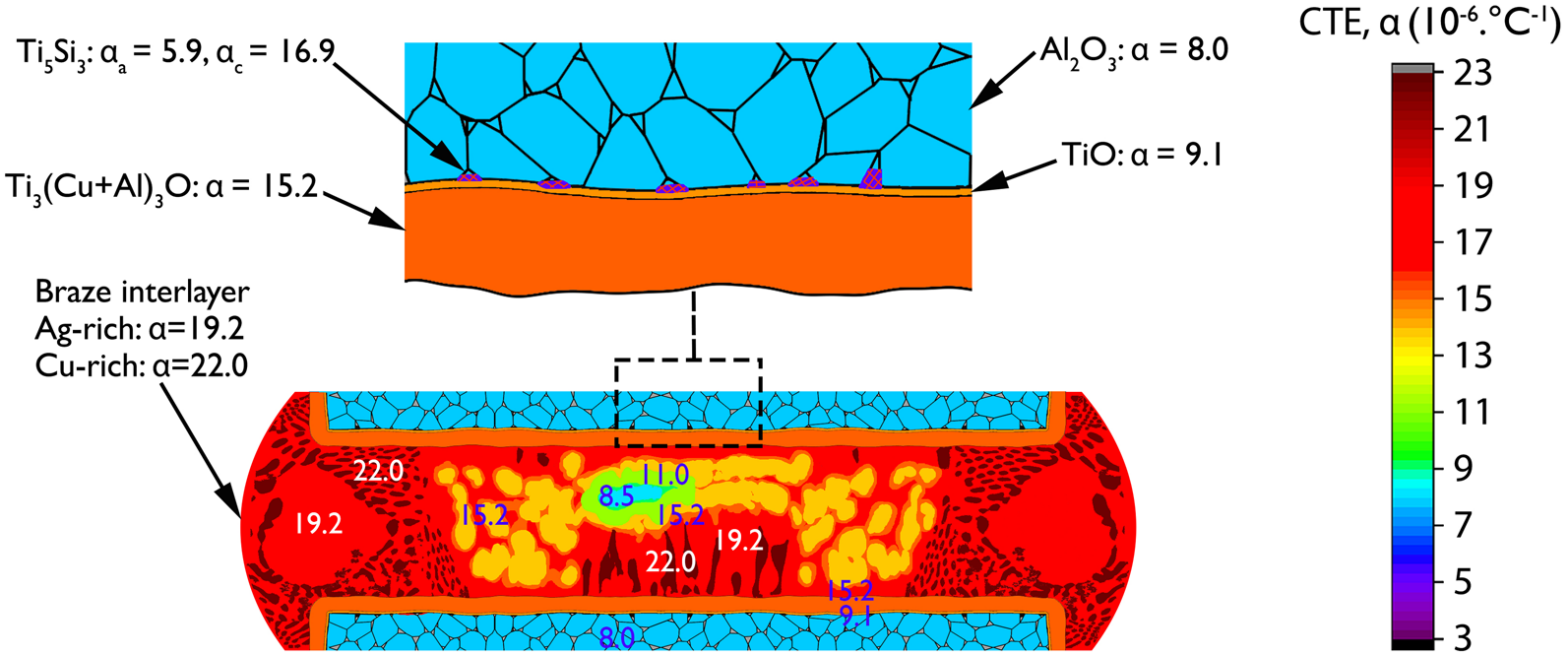
Schematic of the coefficient of thermal expansion α, (× 10^−6^. °C^−1^) values of phases in an alumina-to-alumina brazed joint made using 96.0 wt.% Al_2_O_3_ and a 150 µm thick Ticusil braze preform, following a brazing experiment at 850 °C for 10 minutes.

The regular spatial distribution of the Ti_5_Si_3_ phase at locations of the interface where the triple pocket grain boundary regions of the 96.0 wt.% Al_2_O_3_ surface intersected with the Ti-rich reaction layers may have led to an improvement in the gradation of the coefficient of thermal expansion (CTE) across the joint interface. The Ti_3_(Cu+Al)_3_O layer, with a CTE value of 15.2 × 10^−6^. °C^−1^, introduces a gradual transition in the CTE across the joint interface between alumina, which has a relatively lower CTE value of 8.1 to 8.5 × 10^−6^. °C^−1^, and the braze interlayer, in which the Ag-rich and Cu-rich phases have CTE values of 19.2 × 10^−6^. °C^−1^ and 22.0 × 10^−6^. °C^−1^, respectively. The CTE of a Ti_5_Si_3_ crystal lattice exhibits high anisotropy, with CTE values of 5.9 × 10^−6^. °C^−1^ along the a-axis and 16.9 × 10^−6^. °C^−1^ along the c-axis^43^. The formation of Ti_5_Si_3_ reaction products may have made the residual stress distribution at the joint interface relatively complex. Secondary phase interaction may have further graded the CTE transition across the joint interface leading to improvements in both the residual stress state of the joints and the joint strength (Fig. 10).

A second potential mechanism may be that the regular spatial distribution of Ti_5_Si_3_ compounds at locations of the interface where the triple pocket grain boundary regions of the 96.0 wt.% Al_2_O_3_ surface intersected with the Ti-rich reaction layers provided a nanostructured interlocking mechanism. This may be similar to the way in which ~20 nm thick Ti_5_Si_3_ dendrites were suggested to improve the strengths of glass-to-Ti-6Al-4V joints^44^. Further, the formation of these Ti_5_Si_3_ phases may have provided additional chemical bonding at the interfaces of 96.0 wt.% Al_2_O_3_ braze joints, enabling relatively higher levels of both, thermally induced residual stresses and applied stresses, to be transferred into the relatively ductile matrix of the braze interlayer. Secondary phase interaction, therefore, may have provided a nanostructured interlocking mechanism which led to the improvement in joint strength observed.

## Conclusions

In the alumina/Ag-Cu-Ti joining system a wide range of alumina ceramics have been selected and these alumina ceramics have predominantly been solid-state sintered. The presence of Si at the interfaces of brazed joints made using liquid phase sintered alumina ceramics and Ag-Cu-Ti braze alloys has previously been observed using EDX techniques in the literature^8,16,28^. The formation and positioning of Ti-Si phases at the joint interface and their effect on joint strength, however, has not previously been reported in the literature. It is hereby reported that alumina-to-alumina brazed joints made using liquid phase sintered 96.0 wt.% Al_2_O_3_ ceramics can achieve superior joint performance in comparison to brazed joints made using solid-state sintered 99.7 wt.% Al_2_O_3_ ceramics, formed under the same brazing conditions. The formation and regular spatial distribution of Ti_5_Si_3_ at locations where the triple pocket grain boundary regions of a 96.0 wt.% Al_2_O_3_ surface intersects with the Ti-rich reaction layers may, (i) benefit the residual stress distribution at the joint interface by enabling an improved gradation in the coefficient of thermal expansion across the joint interface, and (ii) provide a nanostructured interlocking mechanism through additional chemical bonding, enabling greater load transfer to the relatively ductile braze interlayer. It is proposed, therefore, that due to the presence of a Si-rich secondary phase, liquid phase sintered alumina ceramics, which are relatively cheaper to manufacture than solid-state sintered alumina ceramics, can be more suitable for achieving high-strength brazed joints.

## Methods

Two commercially available grades of polycrystalline alumina, Dynallox 96 (96.0 wt.% Al_2_O_3_) and Dynallox 100 (99.7 wt.% Al_2_O_3_), manufactured by CoorsTek Ltd, Crewe, UK, were used to produce test bars of two different geometries. Standard test bars had dimensions of 90 mm × 8 mm × 6 mm and short test bars, prepared for brazing experiments, had dimensions of 45 mm × 8 mm × 6 mm. All test bars were ground and chamfered in the same way, according to ASTM C1161-13^45^. The commercially available braze alloy Ticusil® was obtained as 150 µm foil from Wesgo Ceramics GmbH (Morgan Advanced Materials plc), Erlangen, Germany.

Alumina-to-alumina brazed joints, whereby short test bars were brazed to themselves, were prepared by first arranging short test bars in a butt-joint configurations. Several of these joint assemblies, aligned vertically, were supported in a bespoke stainless steel fixture, designed to allow uniform heating of each joint interface during brazing. The brazing fixture and fittings were spray coated using the boron nitride based LOCTITE SF 7900 also known as AERODAG CERAMSHIELD. This was to act as a stop-off, protect the brazing fixture during brazing, and prevent any undesirable effects such as the alumina test bars reacting with, or becoming clamped in, the stainless steel brazing fixture.

For each joint assembly, a braze preform was placed between the faying surfaces of two short test bars and five joints were produced in each brazing cycle. The braze preforms had dimensions of 7 × 5 mm and were mechanically punched from the 150 µm thick Ticusil braze foil. No additional load other than the self-weight of the upper short test bar in each joint assembly was applied. Prior to each brazing cycle, the short test bars, braze preforms, and the brazing fixture and fittings, were all ultrasonically cleaned in acetone for 15 minutes.

Brazing was performed in a Vacuum Generation Ltd vacuum furnace, at a pressure of 1 × 10^−5^ mbar. Thermocouples, placed in the brazing fixture, were used to control the furnace temperature. A peak brazing temperature of 850 °C and a brazing time of 10 minutes followed a 10 minute isothermal soak at 750 °C. This enabled the temperatures of the brazing fixture and the joint assemblies to homogenise before the peak brazing temperature was reached. The heating and cooling rates were 10 °C/min and 5 °C/min, respectively. Alumina crucibles containing Ti granules were used as getters to reduce the partial pressure of oxygen in the vacuum furnace during brazing.

Both the standard test bars and brazed joints were mechanically tested using four-point bend testing, which was conducted at ambient room temperature according to the ASTM C1161-13^45^.

Polished sections of both, the standard alumina test bars and cross-sections of the brazed joints, were first studied using a Zeiss ΣIGMA™ scanning electron microscope (SEM). Electron probe microanalysis (EPMA) was performed using a Cameca SX-100 with five wavelength dispersive spectroscopy detectors and an FEI Nova 600 Nanolab Dualbeam FIB/FEG-SEM was used to prepare thin lamella specimens for transmission electron microscopy (TEM) analysis using the *in-situ* ‘lift-out’ method. These specimens were studied using a JEOL 2100 field emission gun TEM with scanning-TEM (STEM) capabilities set to an operating voltage of 200 kV. TEM electron micrograph and electron diffraction micrograph images were acquired using a Gatan Orius CCD camera, while STEM images were acquired using Jeol bright-field and/or dark-field detectors and Gatan Digiscan image acquisition software. Image processing of micrographs was carried out using Gatan Microscopy Suite. Energy dispersive X-ray (EDX) analysis and STEM-EDX mapping was carried out using a Thermo Scientific Noran 7 silicon drift detector EDX system coupled with NSS software.

### Data Availability

All metadata pertaining to this work can be accessed via the following link: 10.17633/rd.brunel.5812995.

## References

[1] Locatelli MR, Dalgleish BJ, Nakashima K, Tomsia AP, Glaeser AM (1997). New approaches to joining ceramics for high-temperature applications. Ceram. Int..

[2] Bahrani AS (1992). The joining of ceramics. Int. J. Joining Mater..

[3] Gauthier, M. M. Joining in *Engineered materials handbook desk edition* 846–864 (ASM International, 1995).

[4] Fernie JA, Drew RL, Knowles KM (2009). Joining of engineering ceramics. Int. Mater. Rev..

[5] Eustathopoulos N (1998). Dynamics of wetting in reactive metal/ceramic systems. Acta Mater..

[6] Mohammed Jasim K, Hashim FA, Yousif RH, Rawlings RD, Boccaccini AR (2010). Actively brazed alumina to alumina joints using CuTi, CuZr and eutectic AgCuTi filler alloys. Ceram. Int..

[7] Valette C, Devismes M, Voytovych R, Eustathopoulos N (2005). Interfacial reactions in alumina/CuAgTi braze/CuNi system. Scr. Mater..

[8] Vianco PT, Stephens JJ, Hlava PF, Walker CA (2003). Titanium scavenging in Ag-Cu-Ti active braze joints. Weld. J..

[9] Shiue RK, Wu SK, Wang JY (2000). Microstructural evolution at the bonding interface during the early-stage infrared active brazing of alumina. Metall. Mater. Trans. A.

[10] Peytour C, Barbier F, Revcolevschi A (1990). Characterization of ceramic/TA6V titanium alloy brazed joints. J. Mater. Res..

[11] Voytovych R, Robaut F, Eustathopoulos N (2006). The relation between wetting and interfacial chemistry in the CuAgTi/alumina system. Acta Mater..

[12] Stephens JJ, Hosking FM, Headley TJ, Hlava PF, Yost FG (2003). Reaction layers and mechanisms for a Ti-activated braze on sapphire. Metall. Mater. Trans. A.

[13] Hahn S, Kim M, Kang S (1998). A study of the reliability of brazed Al_2_O_3_ joint systems. IEEE Trans. Compon., Packag., Manuf. Technol., Part C.

[14] Lee WC, Kwon OY, Kang CS (1995). Microstructural characterization of interfacial reaction products between alumina and braze alloy. J. Mater. Sci..

[15] Ichimori T, Iwamoto C, Tanaka S (1999). Nanoscopic analysis of a Ag-Cu-Ti/sapphire brazed interface. Mater. Sci. Forum.

[16] Ali M, Knowles KM, Mallison PM, Fernie JA (2015). Microstructural evolution and characterisation of interfacial phases in Al_2_O_3_/Ag-Cu-Ti/Al_2_O_3_ braze joints. Acta Mater..

[17] Byun W, Kim H (1994). Variations of phases and microstructure of reaction products in the interface of Al_2_O_3_/Ag-Cu-Ti joint system with heat treatment. Scr. Metall. Mater..

[18] Kelkar GP, Carim AH (1993). Synthesis, properties, and ternary phase stability of M_6_X compounds in the Ti-Cu-O system. J. Am. Ceram. Soc..

[19] Cho HC, Yu J (1992). Effects of brazing temperature on the fracture toughness of Al_2_O_3_/Ag-Cu-0.5Ti joints. Scr. Metall. Mater..

[20] Loehman RE, Tomsia AP (1992). Reactions of Ti and Zr with AlN and Al_2_O_3_. Acta Metall. Mater..

[21] Suenaga S, Nakahashi M, Maruyama M, Fukasawa T (1997). Interfacial reactions between sapphire and silver-copper-titanium thin film filler metal. J. Am. Ceram. Soc..

[22] Kozlova O (2010). Brazing copper to alumina using reactive CuAgTi alloys. Acta Mater..

[23] Conquest, D. B. *Brazing of alumina for high temperature applications* (University of Cambridge, 2003).

[24] Hao H, Jin Z, Wang X (1994). The influence of brazing conditions on joint strength in Al_2_O_3_/Al_2_O_3_ bonding. J. Mater. Sci..

[25] Ning H (2003). Joining of sapphire and hot pressed Al_2_O_3_ using Ag70.5Cu27.5Ti2 brazing filler metal. Ceram. Int..

[26] Carim AH, Mohr CH (1997). Brazing of alumina with Ti_4_Cu_2_O and Ti_3_Cu_3_O interlayers. Mater. Lett..

[27] do Nascimento RM, Martinelli AE, Buschinelli AJA (2003). Review article: Recent advances in metal-ceramic brazing. Ceramica.

[28] Kassam TA, Nadendla HB, Ludford N, Buisman I (2016). The effect of post-grinding heat treatment of alumina and Ag-Cu-Ti braze preform thickness on the microstructure and mechanical properties of alumina-to-alumina brazed joints. J. Mater. Eng. Perform..

[29] Morrell R. *Handbook of properties of technical & engineering ceramics, Part 2* (H.M.S.O, 1985).

[30] Lee, W. H. *et al*. Self-consolidation mechanism of nanostructured Ti_5_Si_3_ compact induced by electrical discharge. *Sci. World J*. **7** (2015). 10.1155/2015/815084 PMC4389978 25884039

[31] Kang S, Selverian JH (1992). Interactions between Ti and alumina-based ceramics. J. Mater. Sci..

[32] Tamai T, Naka M (1996). Ti effect on microstructure and strength of Si_3_N_4_/Si_3_N_4_ and SiC/SiC joints brazed with Cu-Ag-Ti filler metals. J. Mater. Sci. Lett..

[33] Liu Y, Huang Z, Liu X (2010). Reaction layer microstructure of SiC/SiC joints brazed by Ag-Cu-Ti filler metal. Key Eng. Mater..

[34] Tillman W, Lugscheider E, Xu R, Indacochea JE (1996). Kinetic and microstructural aspects of the reaction layer at ceramic/metal braze joints. J. Mater. Sci..

[35] Asthana R, Singh M, Martinez-Fernandez J (2013). Joining and interface characterization of *in-situ* reinforced silicon nitride. J. Alloys Compd..

[36] Naka M, Tanaka T, Okamoto I (1985). Joining of silicon nitride to metals or alloys using amorphous Cu-Ti filler metal. Trans. Japan Weld. Res. Inst..

[37] Loehman RE, Tomsia AP, Pask JA, Johnson SM (1990). Bonding mechanisms in silicon nitride brazing. J. Am. Ceram. Soc..

[38] Suganuma K, Miyamoto Y, Koizumi M (1988). Joining of ceramics and metals. Ann. Rev. Mater. Sci..

[39] Singh M, Martínez-Fernández J, Asthana R, Ramirez Rico J (2012). Interfacial characterization of silicon nitride/silicon nitride joints brazed using Cu-base active metal interlayers. Ceram. Int..

[40] Singh M, Asthana R, Varela FM, Martínez-Fernández J (2011). Microstructural and mechanical evaluation of a Cu-based active braze alloy to join silicon nitride ceramics. J. Eur. Ceram. Soc..

[41] Xian AP, Si ZY (1990). Joining of Si_3_N_4_ using Ag57Cu38Ti5 brazing filler metal. J. Mater. Sci..

[42] Urai S, Naka M (1999). Effect of Sn addition to Cu-Ti filler metals on microstructure and strength of SiC/SiC joint. Trans. Japan Weld. Res. Inst..

[43] Rodrigues G, Nunes CA, Suzuki PA, Coelho GC (2006). Thermal expansion of the Ti_5_Si_3_ and Ti6Si2B phases investigated by high-temperature X-ray diffraction. Intermetallics.

[44] Oku T (2001). Structural characterization of the metal/glass interface in bioactive glass coatings on Ti-6Al-4V. J. Mater. Sci.: Mater. Med..

[45] ASTM Standards C1161-13: Standard test method for flexural strength of advanced ceramics at ambient temperature 1–19 (ASTM, 2013).

[46] Rana A. *Comparative techniques of joining alumina* (Sheffield Hallam University, 2006).

[47] Yang J, Fang H, Xin W (2005). Al_2_O_3_/Al_2_O_3_ joint brazed with Al_2_O_3_-particulate-contained composite Ag-Cu-Ti filler material. J. Mater. Sci. Technol..

[48] Yang J, Fang H, Xin W (2002). Effects of Al_2_O_3_-particulate-contained composite filler materials on the shear strength of alumina joints. J. Mater. Sci. Technol..

[49] Hosking FM (2000). Microstructural and mechanical characterization of actively brazed alumina tensile specimens. Weld. J..

[50] Voytovych R, Ljungberg LY, Eustathopoulos N (2004). The role of adsorption and reaction in wetting in the CuAg-Ti/alumina system. Scr. Mater..

[51] Mandal S, Rao V, Ray AK (2004). Characterization of brazed joint interface between Al_2_O_3_ and (Ag-Cu-Ti). J. Mater. Sci..

[52] Mondal, S., Gunjan, M. K., Ghosh, R. N. & Ray, A. K. Alumina-alumina joining by Ag-Cu-Ti active filler alloy in *Frontiers of Welding Science and Technology* (International Institute of Welding, 2005).

[53] Rijinders MR, Peteves SD (1999). Joining of alumina using a V-active filler metal. Scr. Mater..

[54] Heikinheimo, L. S. K., Siren, M. J., Kauppinen, K. P. & Auerkari, P. M. S. Performance of alumina-alumina and alumina-metal joints in *Mis-matching of interfaces and welds* (eds Schwalbe, K. H. & Kocak, M.) 451–462 (GKSS Research Centre Publications, 1997).

[55] Hao H, Wang Y, Jin Z, Wang X (1997). Interfacial morphologies between alumina and silver-copper-titanium alloy. J. Mater. Sci..

[56] Santella ML, Horton JA, Pak JJ (1990). Microstructure of alumina brazed with a silver-copper-titanium alloy. J. Am. Ceram. Soc..

[57] Moorhead, A. J. & Santella, M. L. The effect of interfacial reactions on the mechanical properties of oxide ceramic brazements in *High Technology Joining* (BNF Metals Technology Centre, 1987).

[58] Moorhead, A. J., Henson, H. M. & Henson, T. J. The role of interfacial reactions on the mechanical properties of ceramic brazements in *Ceramic microstructures ‘86*: Role *of interfaces* (eds Pask, J. A. & Evans, A. G.) 949–958 (Plenum Press, 1987).

[59] Mizuhara H, Mally K (1985). Ceramic-to-metal joining with active brazing filler metal. Weld. J..

[60] Lin K, Singh M, Asthana R (2014). Interfacial chacterization of alumina-to-alumina joints fabricated using silver-copper-titanium interlayers. Mater. Charact..

[61] Ghosh S (2012). Characterization of alumina-alumina/graphite/monel superalloy brazed joints. Ceram. Int..

[62] Kozlova O, Voytovych R, Eustathopoulos N (2011). Initial stages of wetting of alumina by reactive CuAgTi alloys. Scr. Mater..

[63] Asthana R, Singh M (2008). Joining of partially sintered alumina to alumina, titanium, hastealloy and C-SiC composite using Ag-Cu brazes. J. Eur. Ceram. Soc..

[64] Kar A, Mandal S, Venkateswarlu K, Ray AK (2007). Characterization of interface of Al_2_O_3_-304 stainless steel braze joint. Mater. Charact..

[65] Kar, A., Mandal, S., Rathod, S. & Ray, A. K. Effect of Ti diffusivity on the formation of phases in the interface of alumina-alumina brazed with 97(Ag40Cu03Ti) filler alloy in *Brazing and Soldering**2006**: Proceedings of the 3*^*rd*^*International Brazing and Soldering Conference* (eds Stephens, J. J. & Weil, K. S.) 219–225 (ASM International, 2006).

[66] Carter, E. *The effect of heat treatment time on the structure and mechanical properties of Ag-Cu-Ti based brazed alumina joints* (Sheffield Hallam University, 2004).

[67] Lin CC, Chen RB, Shiue RK (2001). A wettability study of Cu/Sn/Ti active braze alloys on alumina. J. Mater. Sci..

[68] Paulasto M, Kivilahti J (1998). Metallurgical reactions controlling the brazing of Al_2_O_3_ with Ag-Cu-Ti filler alloys. J. Mater. Res..

[69] Bang KS, Liu S (1994). Interfacial reaction between alumina and Cu-Ti filler metal during reactive metal brazing. Weld. J..

[70] Moorhead AJ, Simpson W (1993). Effect of surface preparation on strength of ceramic joints brazed with active filler metal. Ceram. Trans..

[71] Moorhead AJ, Kim HE (1991). Oxidation behaviour of titanium-containing brazing filler metals. J. Mater. Sci..

[72] Moorhead AJ (1987). Direct brazing of alumina ceramics. Adv. Ceram. Mater..

[73] Ali M, Knowles KM, Mallinson PM, Fernie JA (2016). Interfacial reactions between sapphire and Ag-Cu-Ti-based active braze alloys. Acta Mater..

[74] Yadav DP (2014). Study on vacuum brazing of high purity alumina for application in proton synchrotron. Mater. Des..

[75] Yang M, Lin T, He P (2012). Microstructure evolution of Al_2_O_3_/Al_2_O_3_ joint brazed with Ag-Cu-Ti  +  B  +  TiH_2_ Composite filler. Ceram. Int..

[76] Janickovic D, Sebo P, Duhaj P, Svec P (2001). The rapidly quenched Ag-Cu-Ti ribbons for active joining of ceramics. Mater. Sci. Eng. A..

